# Does Wind Disrupt Overwintering Monarch Butterfly Clusters? An Observational Study of Western Monarchs

**DOI:** 10.3390/insects17100993

**Published:** 2026-09-25

**Authors:** Kyle Nessen, Peter C. Ibsen, Jay E. Diffendorfer, Francis X. Villablanca

**Affiliations:** 1 Biological Sciences Department, California Polytechnic State University, San Luis Obispo, CA 93407, USA; fvillabl@calpoly.edu; 2 U.S. Geological Survey, Geosciences and Environmental Change Science Center, Denver, CO 80225, USA; pibsen@usgs.gov (P.C.I.); jediffendorfer@usgs.gov (J.E.D.)

**Keywords:** clustering behavior, conservation, *Danaus plexippus*, habitat management, microclimate, overwintering, thermoregulation, western monarch butterfly, wind disruption

## Abstract

Wind protection is a central focus of western monarch butterfly (*Danaus plexippus*) overwintering habitat conservation. The Wind Disruption Hypothesis guiding management decisions predicts that winds at or above 2 m/s disrupt clusters and that cluster abundance decreases as wind speed increases. At two California groves during the 2023–2024 overwintering season, we paired time-lapse estimates of visible cluster size with nearby measurements of maximum wind gust. Stronger gusts were not consistently followed by decreases over 30-min or next-day response windows. In the 30-min analysis, the wind association depended on approximate local temperature and whether visible butterflies were in direct sunlight. Stronger gusts were associated with declines under cool, sunny conditions and increases under warm, sunny conditions. No clear wind association was detected when no visible butterflies were in direct sunlight. The next-day analysis likewise indicated that the wind association varied with sun exposure. Nearby maximum gusts did not measure sustained exposure at butterfly positions, so this was not an exact test of the historical threshold. The results did not support the predicted consistent decline with increasing wind under the monitored conditions. Physiological responses to interacting wind, ambient temperature, and solar exposure offer a plausible hypothesis for future research.

## Simple Summary

Wind speeds at or above 2 m/s have long been proposed to disrupt overwintering monarch butterfly clusters, with cluster size expected to decrease consistently as wind speed increases. We monitored visible clusters and nearby wind at two California groves during one overwintering season. Stronger wind gusts were not consistently followed by decreases in visible cluster size. Instead, the association between wind and cluster change depended on temperature and whether butterflies were visible in direct sunlight. Under cool, sunny conditions, stronger gusts were associated with decreases in visible cluster size. Under warm, sunny conditions, they were associated with increases. When no visible butterflies were in direct sunlight, we found no clear association between wind and cluster change. These results did not support the predicted consistent decline in cluster size with increasing wind under the monitored conditions. The observed patterns may reflect how wind, temperature, and sunlight jointly affect butterfly body temperature and energy use.

## 1. Introduction

Each autumn, western monarch butterflies (*Danaus plexippus*) migrate from breeding areas across western North America to tree groves along the California coast, where they form dense overwintering aggregations [1,2,3]. Monarch aggregations often recur in the same grove areas and trees across years, yet can shift within a season as conditions change [4,5]. Because wind and other abiotic conditions strongly shape invertebrate activity and habitat use, local microclimate offers a plausible explanation for this combination of predictable habitat use and short-term movement [6,7,8,9,10,11]. Researchers have therefore long sought to identify the environmental conditions that distinguish monarch aggregation locations within overwintering groves.

Early research on monarch overwintering ecology in Mexico identified temperature, solar radiation, moisture, and protection from storms as conditions that could influence survival and activity [12,13,14]. Building on this work, overwintering studies in central California compared the microenvironment around occupied and unoccupied trees within western monarch overwintering groves [4,15]. Monarch aggregations were associated with trees and grove locations characterized by low wind, filtered sunlight, cool temperatures, and available moisture. Movements among trees were also interpreted as responses to changing environmental conditions. Together, these observations contributed to the Microclimate Hypothesis, which proposed that monarchs select aggregation locations that provide a suitable combination of wind, temperature, light, and moisture [4,15]. Later accounts placed particular emphasis on wind, describing monarchs being dislodged from branches when wind speeds in groves reached or exceeded 2 m/s, moving to more sheltered portions of a grove, or falling to the ground when temperatures were too low for flight [16,17]. We use the term Wind Disruption Hypothesis for the resulting prediction that winds at or above 2 m/s disrupt monarch aggregations and cause butterflies to relocate or leave a grove.

These interpretations were subsequently incorporated into overwintering habitat assessments and management guidance. Wind speeds at or above 2 m/s were treated as disruptive, not necessarily just at clusters, and wind protection became an important objective of grove management [17,18,19]. Decisions about planting, pruning, and removing trees were therefore often made in response to perceived wind exposure and the need to maintain or restore sheltered conditions [20,21,22]. Although temperature, sunlight, and moisture remained components of the broader Microclimate Hypothesis, wind protection became a major focus of grove management, guided by the specific 2 m/s threshold.

The studies supporting this wind threshold did not directly quantify how monarch clusters responded during measured wind exposure. Instead, wind effects were inferred from comparisons of occupied and unoccupied locations and from later qualitative accounts [4,15,16]. More recent investigations have also complicated the broader Microclimate Hypothesis. Studies across multiple California groves found that temperature, humidity, and light at aggregation locations varied with latitude, local geography, and spatial scale rather than forming a single environmental envelope [23,24]. Those studies did not evaluate butterfly responses to wind, leaving the Wind Disruption Hypothesis unresolved. It therefore remains unclear whether increasing wind consistently reduces cluster size or whether apparent responses to wind may arise through other processes, including possible interactions with temperature and sunlight [12,25,26].

To evaluate the Wind Disruption Hypothesis, we conducted an observational field study at two blue gum eucalyptus groves at Vandenberg Space Force Base during the 2023–2024 overwintering season. Time-lapse imagery provided repeated estimates of visible cluster size, summarized as the Butterfly Index, while nearby sensors recorded maximum wind gusts. We tested whether stronger gusts were followed by consistent reductions in visible cluster size over short and overnight timescales, and whether those reductions increased with wind speed. This paired monitoring evaluated the central prediction that wind disrupts overwintering monarch aggregations.

## 2. Materials and Methods

### 2.1. Study Design and Sites

We conducted an observational field study at Vandenberg Space Force Base (VSFB, 34.7398° N, 120.5725° W) in Santa Barbara County, California. VSFB contains 30 documented monarch overwintering groves, principally in mature blue gum eucalyptus (*Eucalyptus globulus*) stands [27]. Monitoring was attempted during the 2023–2024 and 2024–2025 overwintering seasons at nine of these groves. The analyses use only observations from 17 November 2023 through 4 February 2024 because only two groves produced usable cluster time series. Monarch abundance during 2024–2025 was insufficient for a second season of analysis [27].

Spring Canyon (SC, Xerces Site ID 2712, 34.6315° N, 120.6182° W) is a 2.0-ha riparian grove of mature blue gum eucalyptus reaching approximately 40 m in height. A perennial creek, variable tree spacing, and heterogeneous understory create a structurally diverse grove. UDMH (Xerces Site ID 2822, 34.6719° N, 120.5950° W), named for rocket fuel formerly stored at the nearby facility, is a 5.1-ha site composed of blue gum eucalyptus windrows with scattered low shrubs. Within the analytical season, equipment was repositioned as visible aggregations shifted, producing ten analyzed monitoring periods, hereafter deployments. Eight were at SC and two were at UDMH.

### 2.2. Monitoring System and Deployment Units

**Figure 1:**
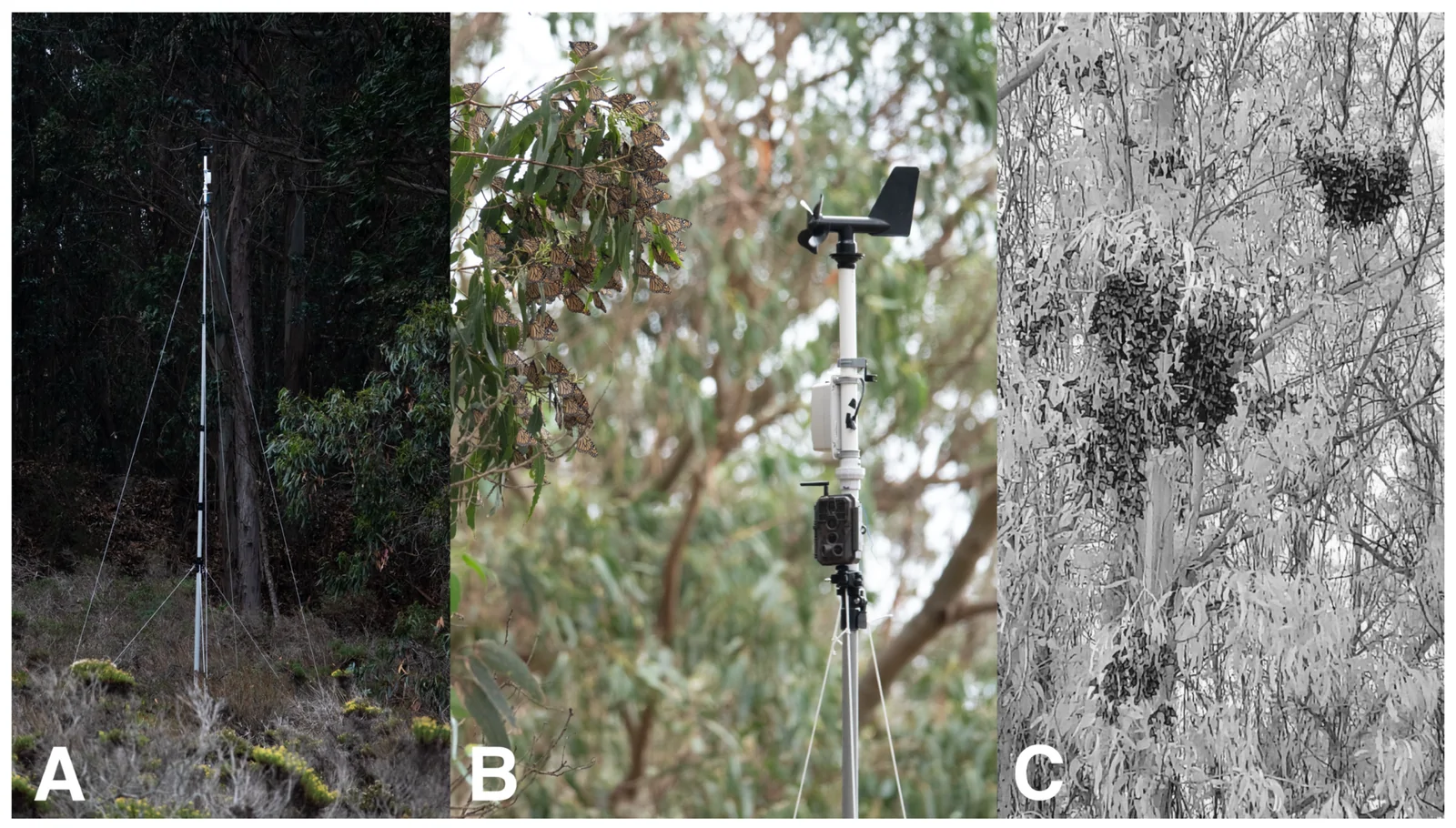
Remote monitoring of overwintering monarch clusters. (**A**) Freestanding guyed telescopic pole deployed within a blue gum eucalyptus grove at Vandenberg Space Force Base. (**B**) Wind logger mounted above a near-infrared-modified trail camera and positioned near a monarch aggregation. (**C**) Representative near-infrared time-lapse image used to calculate the Butterfly Index, in which monarch aggregations appear as dark masses against highly reflective vegetation. Classifications included only butterflies visible within the fixed camera field of view.

We mounted monitoring equipment on freestanding fiberglass telescopic poles with a maximum extension of 15 m (Max-Gain Systems, Inc., Marietta, GA, USA) supported by triangular bases and guy lines (Figure 1). For analyzed deployments, the equipment was positioned 5.4–9.4 m above ground and 4.2–16.5 m horizontally from the monitored cluster. Pole height was selected to approximate the elevation of the visible roost area while maintaining an unobstructed camera view. The guyed poles remained stable and did not visibly move or rotate during included deployments. Cluster height varied during observation periods and was not recorded systematically, so vertical sensor-to-cluster distances could not be calculated. Available placement metadata for each analyzed deployment are summarized in Appendix A.

GardePro E7 and E8 trail cameras (Shenzhen, China) were modified with near-infrared pass filters (>850 nm). Near-infrared imaging rendered clustered butterflies dark against bright eucalyptus foliage, increasing visual contrast [28,29,30]. Cameras operated in time-lapse mode with motion detection disabled and remained fixed within each deployment. Images were acquired at deployment-specific intervals of 5 or 10 min and standardized to a 30-min series by deterministic downsampling. Only daylight images were retained for butterfly classification because passive near-infrared imaging required ambient light to distinguish butterflies reliably from foliage. Cameras were positioned with an unobstructed line of sight to the principal monitored cluster area. Only butterflies visible within the fixed field of view were subsequently classified.

WindLog data loggers (RainWise Inc., Trenton, ME, USA) were installed at pole apices above the cameras. Loggers recorded average wind speed, maximum wind gust, and direction at one-min intervals. Wind records were plotted by deployment and examined for implausible spikes, repeated zeros, and abnormal variance. Where deployments overlapped, concurrently operating loggers were compared to identify gross disagreement in temporal patterns. The only deployment flagged for abnormal sensor behavior contained no butterflies and did not contribute to the analytical datasets. These procedures assessed gross logger performance but did not constitute calibration against a reference sensor or validation of wind exposure at the butterfly location.

Wind direction was not analyzed because the historical prediction evaluated here concerns disruption as a function of wind-speed magnitude rather than direction, and logger compass headings were not considered sufficiently reliable for directional inference after prolonged coastal exposure. In addition, the amount and arrangement of intervening canopy between the poles and clusters varied among deployments, and its effects on local airflow were not measured. A pole heading therefore could not be used to infer directional exposure at butterfly positions. We interpret the logger values as nearby measurements at the pole position, not as demonstrated wind exposure at every butterfly position.

A deployment was an uninterrupted monitoring period defined by one pole location, fixed camera configuration, cluster view, and associated wind logger. Camera servicing or repositioning ended one deployment and began another. Deployments are observation and time-series units, not independent biological populations. Some deployments may have monitored the same aggregation or some of the same butterflies.

### 2.3. Image Classification and Derived Variables

Human labelers were trained using an illustrated online protocol (https://kylenessen.github.io/monarch_trailcam_classifier/, accessed on 3 September 2026) with example images and detailed classification criteria. We used the associated custom Electron application to overlay each image with a deployment-specific grid. Grid dimensions were selected according to camera distance and image resolution and remained fixed within a deployment. Labelers classified each cell as containing 0, 1–9, 10–99, or 100–999 butterflies. They also marked occupied cells receiving clearly distinguishable direct rather than diffuse illumination.

Classifications described only butterflies visible in the two-dimensional image. Labelers did not estimate butterflies obscured by foliage or other butterflies, cluster depth, or butterflies outside the field of view. Branch movement was present and was addressed by manually classifying every retained image under the same protocol. This process could still introduce short-term classification noise when a visible group fell near a category boundary. Each deployment was classified by one labeler, and no image set received independent replicate classifications. Formal inter-observer agreement therefore could not be estimated. Classifications were reviewed for mislabeled cells, incorrect category assignments, and inconsistent application of the protocol, with corrections communicated across the labeling team.

We calculated a Butterfly Index (BI) for each image:

(1) $$\text{Butterfly}\;\text{index}=\sum_{i}\rho_{i}\times C_{i}$$

where $\rho_{i}$ is the number of cells assigned to category *i* and $C_{i}$ is its numerical category value. We used the lower bounds 1, 10, and 100 as a conservative numerical encoding of the three occupied categories. BI is therefore an index of visible cluster size rather than a count of individually identified butterflies. Using the same category values, we calculated sun-exposed BI as the BI subtotal from occupied cells classified as receiving direct sunlight. Sun-exposed BI is an abundance index for sunlit cells, not a physical measurement of irradiance or light intensity.

Temperature values were extracted from camera-image overlays with an optical-character-recognition script and then reviewed and corrected manually. The camera sensors were not independently calibrated against reference temperature loggers. Their readings may reflect camera housing and local exposure as well as ambient air temperature, so we treat temperature as an approximate local measurement. Solar irradiance, humidity, vapor pressure deficit, precipitation, and surface wetness were not measured.

### 2.4. Statistical Analysis

#### 2.4.1. 30-min Response Window

We paired BI at time *t* with BI at t−30 min for each daytime deployment series, allowing a ±5-min tolerance for variation in image timing. Pairs with BI equal to zero at both observations were excluded as uninformative. The resulting dataset contained 1894 pairs across 115 deployment-days. Of these, 1873 pairs came from eight SC deployments and 21 came from two UDMH deployments. We defined the response as $\Delta \mathrm{BI}={\mathrm{BI}}_{t}-{\mathrm{BI}}_{t-30}$ and applied a signed cube-root transformation, $y={\mathrm{sign}\left(\Delta \mathrm{BI}\right)|\Delta \mathrm{BI}|}^{1/3}$, to improve residual normality while preserving the direction of change.

The environmental predictors were maximum gust within the 30-min interval, mean camera temperature between the paired images, and sun-exposed BI at the earlier image. Average sustained speed, modal gust, and gust standard deviation were not included because they were strongly correlated with maximum gust (Pearson r=0.90, 0.86, and 0.73, respectively). BI at the earlier image and minutes since the first daily observation were adjustment covariates. Previous BI accounted for differences in the amount of visible cluster available to change, and time since the first daily observation accounted for systematic daily activity patterns. Previous BI also contributes mathematically to ΔBI, so its coefficient was not interpreted as a biological response.

#### 2.4.2. Next Day Response Window

The response time for wind-associated cluster change was unknown, so we also evaluated change over a window beginning at the previous day’s maximum BI and ending at the current day’s last daytime image. This endpoint approximated the end of the second day’s observation period. We retained deployment-days containing 15–25 daytime images, paired only consecutive calendar days, and excluded pairs with a daily maximum BI of zero on both days. We required at least 95% overall data completeness, calculated as the geometric mean of temperature, wind, and daylight-image coverage. The response was the change in daily maximum BI and was transformed as $y=\mathrm{sign}\left(\Delta {\mathrm{BI}}_{\max}\right)\sqrt{|\Delta {\mathrm{BI}}_{\max}|}$. These criteria produced 96 pairs from six SC deployments. No UDMH deployment contributed a pair after the daily-coverage and consecutive-day criteria were applied.

The environmental predictors were maximum gust during the window, cumulative sun-exposed BI, and three alternative temperature summaries. These were minimum temperature, maximum temperature, and camera temperature at the previous day’s maximum BI. Temperature summaries were evaluated in separate statistical models rather than combined in the same model. Mean window temperature was not analyzed because the window spanned both daytime and nighttime conditions. Cumulative sun-exposed BI was the sum of sun-exposed BI across daylight images within the window and therefore combined exposure duration with the indexed abundance visible in sunlit cells. Previous-day maximum BI and window duration were adjustment covariates. As in the 30-min analysis, the previous BI coefficient was not interpreted as a biological response because the variable also contributes to the response definition.

#### 2.4.3. Model Comparison, Diagnostics, and Sensitivity Analyses

We fitted multiple candidate generalized additive mixed models with mgcv version 1.9.4 in R version 4.6.1. Both response windows used the same environmental hypothesis templates, applied to the window-specific wind, temperature, and sun-exposed BI summaries. The templates represented adjustment covariates only, single environmental predictors, additive effects, linear two-way interactions, a full linear three-way interaction, additive smooths, and tensor interactions. Linear interaction models followed marginality by including their lower-order terms. Smooth candidates included a centered tensor interaction and a hierarchical tensor model. The centered tensor represented a joint wind and sun-exposed BI response after excluding their marginal basis functions. The hierarchical tensor included marginal wind and sun-exposed BI smooths as well as their interaction. The 30-min set contained 17 candidates. The Next Day set contained 35 because each of the nine temperature-dependent templates was evaluated separately with the three temperature summaries.

Every primary 30-min candidate included previous BI and linear minutes since the first daily observation as adjustment covariates. The 30-min sensitivity analyses repeated the complete candidate set after omitting previous BI and after omitting time since the first daily observation. Every primary Next Day candidate included previous-day maximum BI and window duration. Its sensitivity analysis omitted previous-day maximum BI but retained window duration. All 30-min models included random intercepts for deployment and deployment-day and an AR(1) residual correlation structure within deployment-day series. Next Day models included a deployment random intercept and an AR(1) residual correlation structure within deployment series. Observer was not included as a separate effect because each deployment was classified by one observer, so observer did not vary independently of deployment. Because all qualifying Next Day pairs came from Spring Canyon, the deployment effect accounts for repeated observations among deployments within one grove and does not provide grove-level replication.

All candidates were fitted using maximum likelihood and ranked by the small-sample corrected Akaike information criterion, AICc. Models within two AICc units of the minimum were considered similarly supported. Akaike weights were calculated within each primary or sensitivity candidate set. The selected model in each set was refitted using restricted maximum likelihood for coefficient estimation, uncertainty, prediction, and diagnostics. We evaluated residual distributions, residual-versus-fitted and quantile-quantile plots, basis dimensions, and residual autocorrelation. All 121 candidate fits converged without warnings. Candidate structures are provided in Appendix B.1 and Appendix B.2. Complete formulas and rankings accompany the reproducibility materials.

Focused sensitivity analyses evaluated alternative numerical values for the BI categories and mean differences among observers. The 30-min category and observer checks used the three-way interaction model with previous BI but without time since the first daily observation. The Next Day category check used the selected primary model. Complete sensitivity methods and outputs are provided with the reproducibility materials.

## 3. Results

### 3.1. Descriptive Statistics

Monitoring at SC and UDMH produced 2028 daylight observations at 30-min intervals from 17 November 2023 through 4 February 2024, across 115 deployment-days. These images yielded 1894 informative pairs for the 30-min analysis. Descriptive values are reported as means ± standard deviations unless otherwise stated.

Across the 1894 paired intervals, maximum wind gusts ranged from 0 to 12.4 m/s, with a mean of 2.2 ± 1.4 m/s and a median of 2.2 m/s. Across the 2028 unique images, camera temperature ranged from 3.0 to 30.0 °C, with a mean of 14.5 ± 3.9 °C and a median of 14.0 °C. Sun-exposed BI was greater than zero in 30.2% of observations (*n* = 612). Among those observations, sun-exposed BI ranged from 1 to 295, with a median of 7.

**Figure 2:**
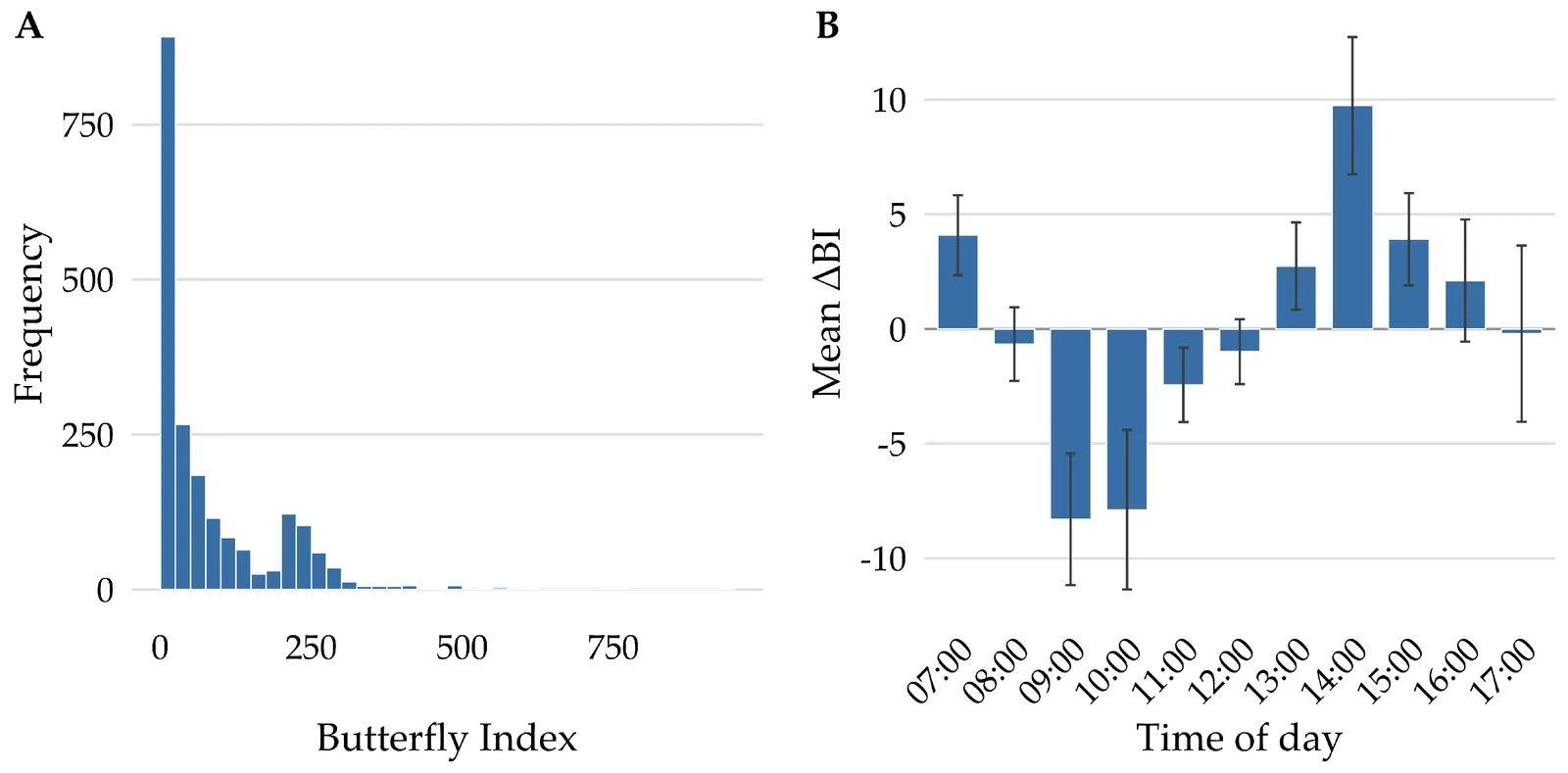
Distribution of visible cluster size and 30-min cluster change. BI is Butterfly Index. (**A**) Histogram of BI across 2028 unique observation images. (**B**) Mean 30-min ΔBI by hour of the ending observation, based on the 1894 paired intervals. Error bars show ±1 SE across deployment-day hourly means.

BI ranged from 0 to 950 per image, with a mean of 80.9 ± 101.4 and a median of 36. Zero values comprised 3.5% of observations (*n* = 71). The BI distribution was strongly right skewed, and the largest mean 30-min decrease occurred at 09:00 and the largest mean increase at 14:00 (Figure 2).

The Next Day Window analysis included 96 consecutive-day pairs with a mean interval of 29.8 h. Previous-day maximum BI ranged from 0 to 770. Changes in daily maximum BI ranged from a loss of 376 to a gain of 464 (mean = −10.5 ± 112.2). Maximum gusts within these intervals ranged from 2.0 to 12.8 m/s (mean = 4.5 ± 1.8 m/s), and cumulative sun-exposed BI ranged from 0 to 1122 (mean = 139.8 ± 206.9).

### 3.2. 30-min Wind Disruption Analysis

To test whether wind was associated with short-term changes in visible cluster size, we compared 17 primary candidate models. The best-supported model included previous BI, time since the first daily observation, and a linear three-way interaction among maximum wind gust, average temperature, and sun-exposed BI. It received nearly all candidate-set support (AICc = 8052.02, Akaike weight > 0.9999). The model containing all three linear two-way interactions ranked second and was 33.80 AICc units higher. The same environmental three-way interaction ranked first when previous BI was omitted and when time since the first daily observation was omitted.

**Figure 3:**
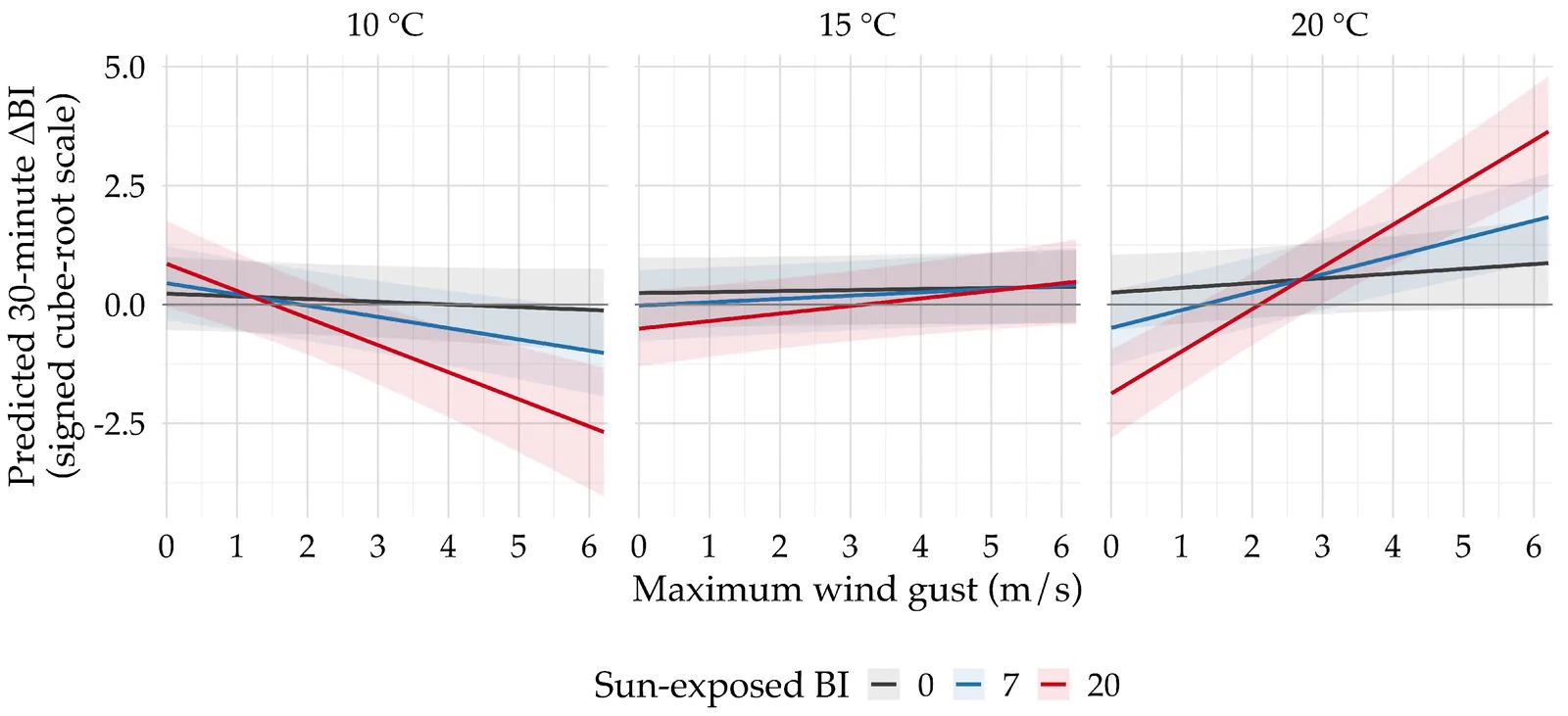
Predicted 30-min change in Butterfly Index (BI) across the three-way interaction among maximum wind gust, temperature, and sun-exposed BI. Predictions are shown on the signed cube-root ΔBI scale, with pointwise 95% confidence intervals shown by shading. Previous BI is held at its median of 37, and time since the first daily observation is held at its median of 300 min. Sun-exposed BI values of 7 and 20 represent the median and 75th percentile among observations with positive sun-exposed BI. Lines extend from 0 to the overall 99th percentile of observed maximum gust, 6.21 m/s. All observations were retained in model fitting.

The fitted relationship between maximum gust and short-term BI change varied with both temperature and sun-exposed BI. Evidence for this joint dependence was strong (three-way interaction, β = 0.00651, SE = 0.00108, *t* = 6.02, p<0.001). The positive interaction term indicates that the conditional wind slope shifted toward more positive values as temperature increased, and that this shift was larger when sun-exposed BI was higher. When sun-exposed BI was zero, wind slopes were not distinguishable from zero at 10, 15, or 20 °C (all p>0.16). At sun-exposed BI values of 7 and 20, slopes were negative at 10 °C (both p<0.001) and positive at 20 °C (both p<0.001). At 15 °C, the slope was not distinguishable from zero at sun-exposed BI of 7 (*p* = 0.063) and was positive at sun-exposed BI of 20 (*p* = 0.018), although predicted changes remained close to zero across the displayed gust range (Figure 3).

Previous BI had a negative coefficient (β = −0.00230, SE = 0.00048, p<0.001), and time since the first daily observation had a small positive coefficient (β = 0.00055, SE = 0.00027, *p* = 0.041). These coefficients were treated as adjustments rather than biological effects. The selected model had an adjusted $R^{2}$ of 0.053 for signed cube-root transformed ΔBI. Residual diagnostics showed modest tail deviations from normality and little remaining temporal autocorrelation.

### 3.3. Next Day Window Analysis

To test whether wind was associated with changes in visible cluster size over a longer response window, we compared 35 primary candidate models. The best-supported model included previous-day maximum BI, window duration, and a centered tensor interaction between maximum wind gust and cumulative sun-exposed BI (AICc = 650.01, Akaike weight = 0.368). A maximum-temperature-only model was also competitive (ΔAICc = 1.89, Akaike weight = 0.143). A linear three-way interaction using temperature at the previous day’s maximum BI ranked third (ΔAICc = 2.99, Akaike weight = 0.082), and the adjustment-only model ranked fifth (ΔAICc = 3.58, Akaike weight = 0.061). The centered tensor interaction remained first in the sensitivity analysis without previous BI, although a three-way interaction using maximum temperature was within one AICc unit (ΔAICc = 0.81).

**Figure 4:**
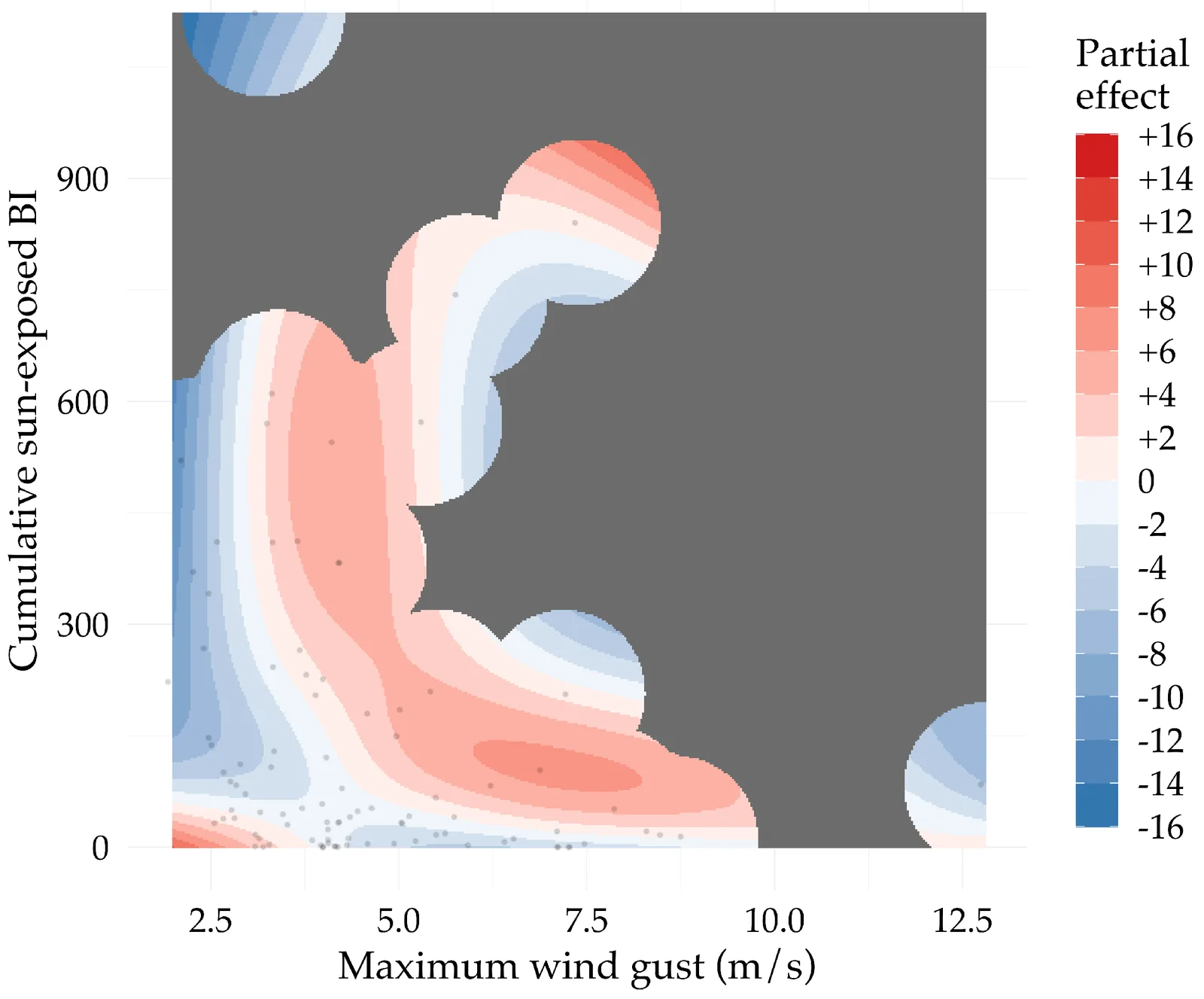
Partial effect of the centered interaction between maximum wind gust and cumulative sun-exposed Butterfly Index (BI) on signed square-root transformed change in daily maximum BI. Positive and negative values indicate point estimates of the interaction’s contribution to the fitted response, not full response predictions. The surface does not show local uncertainty. Jittered points show observed predictor combinations. Gray areas fall outside the supported interpolation region and were not interpreted.

Within the selected model, predicted Next Day Window BI change varied across combinations of maximum wind gust and cumulative sun-exposed BI (edf = 6.68, *F* = 4.10, p<0.001). The interaction’s fitted contribution was positive at low gust speeds and low cumulative sun-exposed BI, negative at similar gust speeds with higher cumulative sun-exposed BI, and positive across parts of the intermediate wind and sun-exposed BI range (Figure 4). Sparse regions outside the reliable interpolation area were not interpreted. Previous-day maximum BI had a negative coefficient (β = −0.0297, SE = 0.0060, p<0.001), whereas window duration was retained as an adjustment but was not supported (β = −0.190, SE = 0.178, *p* = 0.289). Because previous-day maximum BI contributed to the response definition, its coefficient was treated as an adjustment rather than interpreted as a biological effect.

The selected model had an adjusted $R^{2}$ of 0.397 for signed square-root transformed change in daily maximum BI. Residual and basis-dimension diagnostics did not indicate major model violations.

As a focused sensitivity analysis, we refitted the 30-min three-way interaction model without time since the first daily observation and the selected Next Day model after representing the occupied image-cell categories (1–9, 10–99, and 100–999 butterflies) by their lower bounds, rounded geometric midpoints, rounded arithmetic midpoints, or upper bounds. The 30-min three-way interaction and Next Day wind by sun-exposed BI interaction remained supported under all four mappings, and the qualitative 30-min conditional patterns were unchanged. Adding observer to the same 30-min sensitivity model did not improve fit (likelihood-ratio test, *p* = 0.957).

## 4. Discussion

For over three decades, the Wind Disruption Hypothesis has held that winds at or above 2 m/s can physically dislodge overwintering monarchs or cause them to relocate, producing declines in cluster abundance with increasing wind speeds [16,17]. We evaluated this prediction at two overwintering groves during one season by pairing nearby maximum wind gusts with subsequent changes in an image-derived index of visible cluster size (Butterfly Index, BI) over 30-min and next-day response windows. Rather than decreasing consistently as wind speed increased, BI decreased under some combinations of temperature and sun exposure and increased under others.

The 30-min analysis provided the clearest evidence for this conditional relationship. The selected model included a three-way interaction among maximum wind gust, approximate local temperature, and sun-exposed BI. Sun-exposed BI was the portion of visible BI in occupied image cells classified as receiving direct sunlight. When sun-exposed BI was zero, meaning no visible occupied cells were classified as receiving direct sunlight, the conditional association between maximum gust and BI change was not distinguishable from zero at approximate local temperatures of 10, 15, or 20 °C.

When sun-exposed BI was positive, meaning some visible butterflies occupied cells classified as receiving direct sunlight, the association changed with temperature. At 10 °C, increasing gusts were associated with declining BI, and this decline became steeper as sun-exposed BI increased. At 15 °C, BI changed little across the displayed wind range. At 20 °C, fitted BI declined at low gust speeds but increased as gust speeds rose, becoming positive at higher gust speeds. Thus, wind was associated with both decreases and increases in visible cluster size depending on temperature and sun exposure.

The Next Day Window analysis also identified a conditional association between maximum gust and cumulative sun-exposed BI. At low gust speeds, the fitted interaction contribution was positive when cumulative sun-exposed BI was low and negative when cumulative sun-exposed BI was high. Positive contributions also occurred across parts of the intermediate wind and sun-exposure range. This pattern is consistent with a conditional wind association over the longer response window. However, the competitive maximum-temperature-only model and the selected model’s Akaike weight of 0.368 indicate substantial model-selection uncertainty.

Maximum gusts frequently exceeded the proposed 2 m/s threshold while butterflies remained visible. Among paired 30-min observations with butterflies present, 57.9% contained maximum gusts at or above 2 m/s. Every observation period in the Next Day Window analysis contained a recorded maximum gust at or above 2 m/s. These values were one-min maximum gust records measured near the monitored clusters. They were not measurements of sustained exposure above 2 m/s at the butterflies themselves and therefore do not provide an exact test of the historical threshold formulation. Nevertheless, neither response window produced the consistent decline in visible cluster size expected under a simple wind threshold model.

Previous work provides context for why a single-threshold response may be incomplete. The Microclimate Hypothesis has commonly been interpreted as proposing that western monarch aggregations occupy a relatively uniform set of environmental conditions [4,15]. Multiscale studies have instead found that microclimatic attributes associated with aggregations vary with latitude, local geography, and spatial scale [23,24], while light-related habitat features remain associated with aggregation patterns [5,23,31]. These findings question a single uniform microclimatic envelope without establishing one alternative mechanism. Previous physiological research [12,25,26,32] has been suggested as the basis for a mechanistic alternative [23]. Direct tests linking wind exposure, physiological responses, and changes in cluster size are still needed.

Monarch butterfly physiology offers one possible explanation for the conditional relationships observed here. As ectotherms, overwintering monarchs must conserve lipid reserves while maintaining the capacity for essential movement [12,32]. Thoracic temperatures of approximately 12.7 to 16 °C are associated with powered flight, but direct solar exposure can warm monarchs above flight temperature even when ambient conditions are cooler [12]. Solar basking may provide an energetic advantage over shivering, which consumes 25 to 31 times more energy than resting [25]. Under warmer conditions, direct sun can elevate body temperature enough to promote heat-avoidance behavior and additional movement [12,26]. Wind may interact with both processes through convective heat transfer [33,34,35,36].

The 30-min pattern is compatible with this thermoregulatory interpretation. At an approximate local temperature of 10 °C, the negative wind association appeared only when butterflies were visible in sunlight. Solar warming may have allowed butterflies to move under otherwise restrictive ambient conditions, while wind altered the thermal value of exposed positions. At an approximate local temperature of 15 °C, little biologically interpretable change was associated with wind. At an approximate local temperature of 20 °C, the transition from declining BI under low wind to increasing BI under higher wind is compatible with a balance between solar or ambient heat gain and convective cooling. Wind could provide passive cooling under warm, sunny conditions, allowing butterflies to remain at or move into exposed locations.

These interpretations remain hypotheses. The observed declines could represent thermally motivated movement, mechanical disturbance, movement to an unmonitored location, or other processes. Likewise, increasing BI under warm and windy conditions does not demonstrate that butterflies actively sought wind or that convective cooling caused their arrival. We did not measure solar irradiance, thoracic temperature, convective heat transfer, metabolic expenditure, lipid depletion, or movement among roost locations.

We provisionally refer to this framework as the Energetic Constraint Hypothesis. Monarchs are ectotherms that regulate body temperature behaviorally while conserving finite lipid reserves across the overwintering season [12,25,32]. Under this hypothesis, movement and roosting behavior may reflect responses to the energetic consequences of interacting and latitudinally variable environmental conditions, including temperature, solar exposure, wind, and other conditions, rather than a response to any single variable. Future work can evaluate this framework by examining overwintering habitat selection and behavior through a thermoregulatory and energetic lens.

Several features of the study constrain the scope of inference. The data came from two blue gum eucalyptus groves on one military installation during a single overwintering season. Nearly all 30-min observations came from one grove, and only that grove contributed to the Next Day Window analysis. Deployments represented monitoring periods rather than independent biological populations, and some may have included the same aggregation or individual butterflies. The Wind Disruption Hypothesis was formulated when California groves supported much larger aggregations than those observed here [4,15]. In large, multilayered clusters, many butterflies may attach to other butterflies rather than directly to vegetation, so cluster size and configuration could alter responses to wind [37].

The measurements introduce additional uncertainty. Wind was measured near the clusters but was not validated at individual butterfly positions within the canopy. Camera temperatures were not independently calibrated against reference sensors. Sun-exposed BI was a coarse image-based index of visible butterflies in occupied cells classified as receiving direct sunlight, not a direct measurement of solar irradiance. It therefore depended on visible cluster size, butterfly behavior, visibility, and canopy geometry and may be related to the response variable. The guyed poles remained stable and camera views did not visibly rotate during included deployments. Every retained image was classified manually under a shared protocol. Branch movement could occasionally shift visible butterflies among grid cells or across BI category boundaries, introducing short-term classification noise. Consequently, changes in visible BI cannot be interpreted as confirmed arrivals, departures, or physical dislodgment.

Within these constraints, the study does not support a simple formulation in which increasing wind consistently reduces overwintering cluster abundance. The principal finding is that the association between wind and visible cluster change depended on other biologically relevant conditions. An energetic interpretation provides a plausible and testable explanation for that conditionality, but establishing the mechanism will require direct physiological and microclimatic measurements.

## 5. Conclusions

Across the wind conditions measured at two overwintering groves during one season, stronger maximum gusts were not consistently followed by declines in visible monarch cluster size. Instead, the wind association depended on approximate local temperature and direct sun exposure. In the 30-min analysis, stronger gusts were associated with declines under cool, sunny conditions and increases under warm, sunny conditions. No clear wind association was detected when visible butterflies were not in direct sunlight. The next-day analysis likewise showed that the wind association varied with sun exposure.

Together, these results did not support the Wind Disruption Hypothesis predictions that strong winds consistently reduce cluster abundance and that reductions increase with wind speed under the monitored conditions. The conditional patterns could reflect physiological and energetic responses to the combined effects of wind, ambient temperature, and solar exposure. This explanation provides a plausible direction for future research.

## Data Availability

The original photographs, deployment metadata, image-level classification summaries (Butterfly Index, sun-exposed Butterfly Index, and category counts), wind measurements, and reviewed image-derived temperature values are available in the U.S. Geological Survey data release [38] at https://doi.org/10.5066/P13IEKEB. Processed analysis datasets, model formulas, analysis code, and generated model outputs are available at https://github.com/kylenessen/monarch-wind-light-manuscript, accessed on 3 September 2026. The illustrated classification protocol is available at https://kylenessen.github.io/monarch_trailcam_classifier/, accessed on 3 September 2026. The image-classification software, its source code, and the original cell-level classification data used in this study are available at https://github.com/kylenessen/monarch_trailcam_classifier, accessed on 3 September 2026.

## Appendix A. Monitoring Deployment Metadata

Table A1 reports the placement measurements recorded for the ten deployments included in the 30-min analysis. View bearing describes the camera viewing direction, not wind direction.

**Table A1:** Available placement metadata for analyzed monitoring deployments.

ID	Grove	Height (m)	Cluster Distance (m)	Bearing (°)
SC1	Spring Canyon	7.0	4.2	90
SC2	Spring Canyon	6.2	6.8	155
SC4	Spring Canyon	5.6	6.3	335
SC6	Spring Canyon	6.9	12.6	210
SC8	Spring Canyon	6.4	16.5	220
SC9	Spring Canyon	5.4	6.7	100
SC10	Spring Canyon	5.9	7.5	320
SC12	Spring Canyon	7.1	12.6	230
UDMH2	UDMH	8.6	6.8	360
UDMH4	UDMH	9.4	6.2	350

Vertical sensor-to-cluster distance could not be reconstructed because cluster height changed and was not recorded systematically. Intervening vegetation and canopy position were not quantified by deployment. Wind loggers were not independently calibrated against a reference sensor, and wind exposure was not validated at the butterfly locations. These limitations applied to every deployment.

## Appendix B. Candidate Model Strategy

Both analyses used a common set of environmental hypothesis templates. Let *C* denote the required adjustment covariates, *W* maximum gust, *S* sun-exposed BI, and *T* one temperature measure. Linear interactions followed marginality. Thus, a three-way interaction included all associated main effects and two-way interactions. Smooth candidates included both a centered tensor interaction and a hierarchical tensor interaction with marginal smooths.

**Table A2:** Environmental predictors and adjustment covariates in the candidate comparisons.

Role	30-min Window	Next Day Window
*C*	Previous BI and minutes since the first daily observation	Previous-day maximum BI and window duration
*W*	Maximum gust within the paired interval	Maximum gust within the response window
*S*	Sun-exposed BI at the earlier image	Cumulative sun-exposed BI across daylight images
*T*	Mean camera temperature between paired images	Minimum temperature, maximum temperature, or temperature at the previous day’s maximum BI, evaluated in separate candidates

**Table A3:** Shared hypothesis templates. Each temperature-dependent template was fitted once in the 30-min analysis and separately for each of the three Next Day temperature summaries.

ID	Hypothesis	Fixed-Effect Structure
H00	Controls only	*C*
H01	Wind	C+W
H02	Sun-exposed BI	C+S
H03	Additive wind and sun	C+W+S
H04	Linear wind by sun interaction	C+W∗S
H05	Centered tensor interaction	C+ti(W,S)
H06	Additive wind and sun smooths	C+s(W)+s(S)
H07	Hierarchical tensor interaction	C+s(W)+s(S)+ti(W,S)
T01	Temperature	C+T
T02	Additive wind, temperature, and sun	C+W+T+S
T03	Wind by temperature	C+W∗T+S
T04	Wind by sun, with temperature	C+W∗S+T
T05	Temperature by sun, with wind	C+T∗S+W
T06	All linear two-way interactions	C+W∗T+W∗S+T∗S
T07	Full linear three-way interaction	C+W∗T∗S
T08	Additive environmental smooths	C+s(W)+s(T)+s(S)
T09	Smooth wind by sun interaction with temperature	C+s(W)+s(T)+s(S)+ti(W,S)

### Appendix B.1. 30-min Candidate Mapping

The primary 30-min comparison contained the eight H templates and nine T templates, for 17 candidates. The adjustment set *C* was previous BI and linear minutes since the first daily observation. The no-previous-BI sensitivity retained time since the first daily observation, and the no-time sensitivity retained previous BI. Each model included random intercepts for deployment and deployment-day and AR(1) correlation within deployment-day. All 51 fits completed without warnings. The full linear three-way interaction ranked first in all three candidate sets.

Table A4 gives the fixed-effect structure of every primary 30-min candidate. In this table, $C_{30}$ is previous BI plus minutes since the first daily observation, $W_{30}$ is maximum gust within the paired interval, $S_{30}$ is sun-exposed BI at the earlier image, and $T_{30}$ is mean camera temperature between the paired images. Univariate smooths used shrinkage thin-plate regression splines with k=5. Tensor interactions used cubic regression spline marginal bases with k=5 per margin. Every candidate also had the random-effect and correlation structure described above.

**Table A4:** The 17 primary candidates in the 30-min model comparison.

ID	Fixed-Effect Structure
H00	$C_{30}$
H01	$C_{30}+W_{30}$
H02	$C_{30}+S_{30}$
H03	$C_{30}+W_{30}+S_{30}$
H04	$C_{30}+W_{30}*S_{30}$
H05	$C_{30}+ti\left(W_{30},S_{30}\right)$
H06	$C_{30}+s\left(W_{30}\right)+s\left(S_{30}\right)$
H07	$C_{30}+s\left(W_{30}\right)+s\left(S_{30}\right)+ti\left(W_{30},S_{30}\right)$
T01	$C_{30}+T_{30}$
T02	$C_{30}+W_{30}+T_{30}+S_{30}$
T03	$C_{30}+W_{30}*T_{30}+S_{30}$
T04	$C_{30}+W_{30}*S_{30}+T_{30}$
T05	$C_{30}+T_{30}*S_{30}+W_{30}$
T06	$C_{30}+W_{30}*T_{30}+W_{30}*S_{30}+T_{30}*S_{30}$
T07	$C_{30}+W_{30}*T_{30}*S_{30}$
T08	$C_{30}+s\left(W_{30}\right)+s\left(T_{30}\right)+s\left(S_{30}\right)$
T09	$C_{30}+s\left(W_{30}\right)+s\left(T_{30}\right)+s\left(S_{30}\right)+ti\left(W_{30},S_{30}\right)$

### Appendix B.2. Next Day Candidate Mapping

The primary Next Day comparison contained the eight H templates and nine T templates repeated for each of the three temperature summaries, for 35 candidates. The adjustment set *C* was previous-day maximum BI and window duration. The no-previous-BI sensitivity removed previous-day maximum BI from all 35 candidates but retained window duration. Each model included a deployment random intercept and AR(1) correlation within deployment. All 70 fits completed without warnings. The centered wind by sun-exposed BI tensor interaction ranked first in both candidate sets.

Table A5 gives the fixed-effect structure of every primary Next Day candidate. Here, $C_{D}$ is previous-day maximum BI plus window duration, $W_{D}$ is maximum gust within the response window, and $S_{D}$ is cumulative sun-exposed BI across daylight images. The three temperature variables are $T_{\min}$, minimum window temperature, $T_{\max}$, maximum window temperature, and $T_{P}$, temperature at the previous day’s maximum BI. Univariate smooths used shrinkage thin-plate regression splines with k=5. Tensor interactions used cubic regression spline marginal bases with k=5 per margin. Every candidate also had the random-effect and correlation structure described above.

All 121 fits were compared using maximum likelihood and AICc. Akaike weights were calculated separately within each response window and sensitivity framework. The selected formulas were refitted using restricted maximum likelihood. Complete machine-readable formulas, fit outcomes, rankings, and selected-model summaries accompany the analysis code.

**Table A5:** The 35 primary candidates in the Next Day model comparison.

ID	Fixed-Effect Structure
H00	$C_{D}$
H01	$C_{D}+W_{D}$
H02	$C_{D}+S_{D}$
H03	$C_{D}+W_{D}+S_{D}$
H04	$C_{D}+W_{D}*S_{D}$
H05	$C_{D}+ti\left(W_{D},S_{D}\right)$
H06	$C_{D}+s\left(W_{D}\right)+s\left(S_{D}\right)$
H07	$C_{D}+s\left(W_{D}\right)+s\left(S_{D}\right)+ti\left(W_{D},S_{D}\right)$
min-T01	$C_{D}+T_{\min}$
min-T02	$C_{D}+W_{D}+T_{\min}+S_{D}$
min-T03	$C_{D}+W_{D}*T_{\min}+S_{D}$
min-T04	$C_{D}+W_{D}*S_{D}+T_{\min}$
min-T05	$C_{D}+T_{\min}*S_{D}+W_{D}$
min-T06	$C_{D}+W_{D}*T_{\min}+W_{D}*S_{D}+T_{\min}*S_{D}$
min-T07	$C_{D}+W_{D}*T_{\min}*S_{D}$
min-T08	$C_{D}+s\left(W_{D}\right)+s\left(T_{\min}\right)+s\left(S_{D}\right)$
min-T09	$C_{D}+s\left(W_{D}\right)+s\left(T_{\min}\right)+s\left(S_{D}\right)+ti\left(W_{D},S_{D}\right)$
max-T01	$C_{D}+T_{\max}$
max-T02	$C_{D}+W_{D}+T_{\max}+S_{D}$
max-T03	$C_{D}+W_{D}*T_{\max}+S_{D}$
max-T04	$C_{D}+W_{D}*S_{D}+T_{\max}$
max-T05	$C_{D}+T_{\max}*S_{D}+W_{D}$
max-T06	$C_{D}+W_{D}*T_{\max}+W_{D}*S_{D}+T_{\max}*S_{D}$
max-T07	$C_{D}+W_{D}*T_{\max}*S_{D}$
max-T08	$C_{D}+s\left(W_{D}\right)+s\left(T_{\max}\right)+s\left(S_{D}\right)$
max-T09	$C_{D}+s\left(W_{D}\right)+s\left(T_{\max}\right)+s\left(S_{D}\right)+ti\left(W_{D},S_{D}\right)$
prev-T01	$C_{D}+T_{P}$
prev-T02	$C_{D}+W_{D}+T_{P}+S_{D}$
prev-T03	$C_{D}+W_{D}*T_{P}+S_{D}$
prev-T04	$C_{D}+W_{D}*S_{D}+T_{P}$
prev-T05	$C_{D}+T_{P}*S_{D}+W_{D}$
prev-T06	$C_{D}+W_{D}*T_{P}+W_{D}*S_{D}+T_{P}*S_{D}$
prev-T07	$C_{D}+W_{D}*T_{P}*S_{D}$
prev-T08	$C_{D}+s\left(W_{D}\right)+s\left(T_{P}\right)+s\left(S_{D}\right)$
prev-T09	$C_{D}+s\left(W_{D}\right)+s\left(T_{P}\right)+s\left(S_{D}\right)+ti\left(W_{D},S_{D}\right)$
